# Laparoscopic Appendectomy versus Mini-Incision Appendectomy in Patients with Lower Body Mass Index and Noncomplicated Appendicitis

**DOI:** 10.1155/2014/138648

**Published:** 2014-12-14

**Authors:** İsmail Özsan, Türker Karabuğa, Ömer Yoldaş, Özcan Alpdoğan, Ünal Aydın

**Affiliations:** Department of General Surgery, Faculty of Medicine, İzmir University, 35520 Izmir, Turkey

## Abstract

Laparoscopic appendectomy has become favored over open surgical methods for its association with decreased postoperative pain, more rapid return to daily activities, and improved cosmetic results. Mini-incision appendectomy was being performed in our clinic for a long time especially in patients with noncomplicated appendicitis and in patients with appropriate body mass index. Although laparoscopy presents obvious advantages especially for obese patients and young women, with respect to the results of our study, mini-incision appendectomy seems to be an alternative for selected patient groups.

## 1. Introduction

Successful appendectomy was first described by McBurney in 1894 [1], and the open surgical approach remained the gold standard for nearly a century. The lifetime risk of developing appendicitis is between 7 and 9% with evidence of increasing incidence [2, 3].

With the advance of minimal invasive surgery, new approaches for the existing operations have been proposed. Semm first described the laparoscopic approach for acute appendicitis in 1983 [4]. Although there has been a controversy at the beginning, laparoscopic appendicectomy (LA) has become common and an acceptable approach in the management of acute appendicitis [5]. LA has become favored over open surgical methods for its association with decreased postoperative pain, more rapid return to daily activities, and improved cosmetic results. However, the literature has shown the association of laparoscopy with specific adverse events such as increased intra-abdominal abscess and hospital costs [6]. For a long while, we were performing mini-incision appendectomy in patients with appropriate body mass index (BMI) and with noncomplicated acute appendicitis. The present study aimed to compare both laparoscopic and mini-incision appendectomies in terms of operation duration, postoperative complications, length of hospital stay, cost analyses, and cosmetic results.

## 2. Materials and Methods

One hundred and sixty-three consecutive patients underwent either laparoscopic appendectomy (LA) or mini-incision appendectomy (MIA) between 2012 and 2014 in our clinic. The decision of performing LA or MIA was made according to patient and surgeon discretion. Investigation of the patient charts revealed that all of the patients' BMI in MIA group was under 25 kg/m^2^. It was also considered the cut-off point for the LA group.

### 2.1. Data and Patient Selection

**Figure 1:**
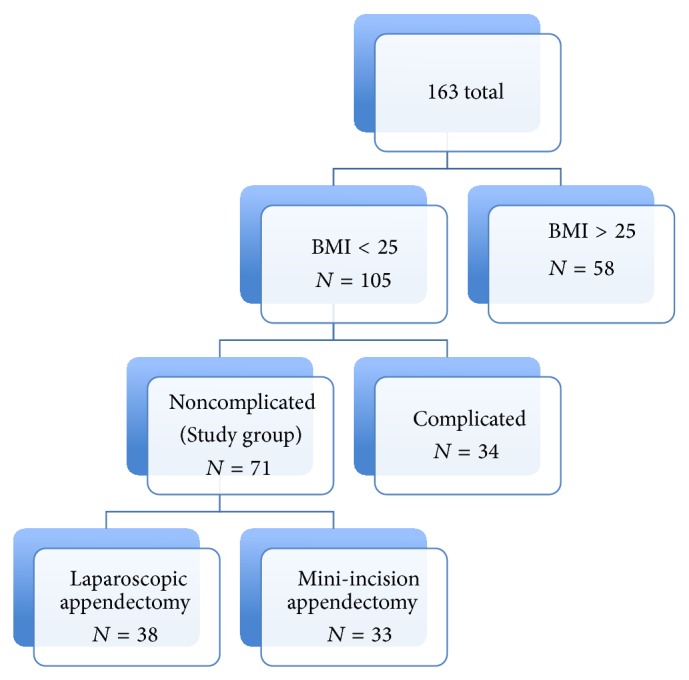
The study group was consisting of the patients whose BMI were lower than 25 kg/m^2^ and who had noncomplicated appendicitis.

A review of charts, which were used routinely in our clinic, was undertaken, with a specific search for documented evidence of each patient's height, weight and also for nonperforated appendicitis. The patients then were categorized according to their BMI. Two groups of patients were identified, those with a BMI lower than 25 kg/m^2^ and those with a BMI higher than 25 kg/m^2^. The first group was then identified to two subgroups, those with perforated or complicated appendicitis and those with nonperforated and noncomplicated appendicitis, and the latter one was the main study group (Figure 1).

### 2.2. Surgical Technique

General anaesthesia was used in LA group. For the laparoscopic approach, the Hasson technique is used and a 10 mm 30° angled scope was used through the 10 mm umbilical trocar, and additional two trocars (10 mm and 5 mm) are placed in the lower abdomen. Mesoappendix is divided by using Ligasure device (Covidien, Colorado, USA) and the appendix stump was clipped by Hem-o-lok polymer ligation clips (Weck/Teleflex). The specimen was removed through the 10 mm suprapubic trocar in a specimen bag. The trocars were removed under direct vision and all trocar sites are closed using 3-0 absorbable monofilament sutures.

For the mini-incision approach, after clinical and radiological diagnosis of acute appendicitis, the most painful point was found on physical examination and marked preoperatively. Regional anaesthesia was used in MIA group. A 1.5 to 2 cm oblique incision from that marked point is used for laparotomy instead of classical Mc Burney incision. Mesoappendix and appendix stump were ligated by 2/0 silk sutures. The stump was routinely inverted with purse string sutures.

The peritoneum was closed by using 3/0 vicryl sutures and the fascia was closed by using nonabsorbable monofilament sutures. The incision was closed by using 4/0 absorbable monofilament suture.

Prospectively collected data of the patients with a clinical diagnosis of acute appendicitis including demographic data (age and gender), preoperative laboratory and radiologic findings, operation type, operation duration, and postoperative course (complications, length of hospital stay, cost analyses, and postoperative cosmetic results) were retrospectively evaluated and the groups were compared retrospectively.

### 2.3. Statistical Analyses

SPSS 16 (SPSS Inc., Chicago, IL, USA) was used for statistical analyses in this study. Chi-square statistical analyses were used for nominal data, student-*t* statistical analyses were used for parametric numerical data, and Mann-Whitney *U* statistical analyses were used for ordinal data and nonparametric numerical data. A *P* value < 0.05 was considered statistically significant.

## 3. Results

The study compared 38 consecutive patients who underwent laparoscopic appendectomy (LA) and 33 patients who underwent mini-incision appendectomy (MIA). The mean ages of the MIA group and LA group were 29.12 ± 8.9 and 32.2 ± 11.4, respectively. The mean BMI of the MIA and LA group was 21.42 ± 2.27 and 21.32 ± 2.38. The groups were similar in age, sex, BMI, and preoperative laboratory findings. In addition to physical and laboratory examination, acute appendicitis was diagnosed by ultrasound only in 47 of the patients while abdominal computed tomography was required for the remaining 24 patients. The mean operation duration for MIA was 24.57 ± 5.87 minutes and 21.34 ± 8.39 minutes for LA. The difference between two groups was not statistically significant (*P* = 0.49). There was no conversion to open procedure in LA group and there was no need to extension of the incision size in MIA group. The length of hospital stay (LOS) was 18.03 ± 3.51 hours in MIA group and 16.92 ± 5.6 hours in LA group. The difference between two groups was not statistically significant (*P* = 0.31). Superficial wound infection occurred in two patients in LA group and 4 patients in MIA group but the difference was not statistically significant (*P* = 0.89). The patients were asked for their cosmetic results by telephone during the study period. They were asked to classify their scar as bad, moderate, good, or excellent. There was no statistically significant difference between two groups (*P* = 0.287). 93.9% of the patients in MIA group described their scar as good or excellent. Total hospital costs of laparoscopic appendectomy were higher by 58.7% when compared to mini-incision appendectomy. There was no identified intra-abdominal abscess in both groups.  Table 1 demonstrates the comparison of the results of the study groups.

## 4. Discussion

Acute appendicitis is a very common pathology encountered in both pediatric and adult patient populations, with a lifetime risk of 7-8% (2). There have been much recent research and debate over the best operative modality for an appendicectomy, despite the laparoscopic approach gaining popularity among general surgeons. The rate of LA between 1998 and 2008 increased from 20.6% to 70.8%, becoming the prevalent approach to treat acute appendicitis since 2005 [7]. In the subset of obese patients, the benefits of laparoscopy are generally more striking, associated with lower risk of intraoperative complications, fewer surgical site infections, and shortened hospital stays [8, 9].

Although the infection of the surgical wound is not a life-threatening condition, it worsens the quality of life in the early postoperative period and prolongs the recovery time. The reduction of wound infection rate is a significant advantage of LA [10]. The extraction of specimen with a bag and through a trocar port rather than directly through the surgical wound as in open procedures can explain this reduction in incidence. Moreover, the smaller size of the laparoscopic incisions reduces the probability of infection, especially in obese patients. Although the number of surgical site infections in MIA group was higher than in LA group, the difference was not significant. This was possibly due to smaller incision sizes of MIA and also due to the selection of the noncomplicated patients in our study.

One of the most known advantages of laparoscopic approach is short lengths of hospital stay. In the present study, the difference was not significant between two groups, possibly due to the smaller sizes of incision and selected patients with lower body mass index and noncomplicated appendicitis. The other known advantage of laparoscopy is good cosmetic results. Patient satisfaction was asked for wound healing and scar tissue in the present study. Although it was not a subjective method to evaluate the cosmetic results of the patients on telephone and only by classifying the scar as bad, moderate, good, and excellent, the difference between two groups was not statistically significant. General anaesthesia is mandatory for laparoscopic procedures, but mini-incision appendectomy provides the option of regional anaesthesia. The use of regional anaesthesia instead of general anaesthesia for mini-incision appendectomy can be recognized as an advantage of MIA.

This study compared the postoperative outcomes and total hospital costs of laparoscopic and mini-incision approaches in the management of noncomplicated appendicitis. We hypothesized that the increase in resources and equipment needed for laparoscopy would result in an overall increase in the cost of hospitalization when LA was used for appendectomy. Wei et al. [11] in their meta-analysis including 8 randomized controlled trials performed an analysis of the costs across different countries and age groups using the hospital cost ratio to compare the total cost of laparoscopic and open appendectomy (OA). The total hospital costs for LA were higher by 11% when compared to OA, but the difference was found to be not statistically significant. According to a Cochrane review published by Sauerland et al. [12], laparoscopy does not show relevant advantages compared to open appendectomy; therefore, indication should be limited to young women and obese patients. Nakhamiyayev et al. [13] and Varela et al. [14] reported that the total hospital costs were comparable between the two procedures or were even lower for the laparoscopic group when the subgroup of obese patients was analyzed.

The main limitations of the present study were those inherent to a retrospective analysis, including lack of prospective validation. Validated prospective collection of patient satisfaction, quality of life, and pain scores also are needed to highlight any improvements in patient-centered outcomes. It should be better to address the comparison of postoperative pain scores between two groups. Thus, further prospective well-designed studies are needed.

Laparoscopic approach presents obvious advantages in some of patient groups such as obese patients, young women patients having a suspect of other diagnoses with or without acute appendicitis, and patients with perforated appendicitis. But in developing countries, total hospital costs are still a serious problem. In conclusion with respect to the results of the present study, mini-incision appendectomy seems to be an alternative for selected patients with lower body mass index and noncomplicated appendicitis.

## Figures and Tables

**Table 1:** Comparison of MIA (mini-incision appendectomy) and LA (laparoscopic appendectomy) according to postoperative complications, cosmetic results, and total hospital costs. The results showed no significant difference between MIA and LA, except total hospital costs. Total hospital costs of LA were higher by 58.7% when compared to MIA.

	LA (*n* = 38)	MIA (*n* = 33)	*P*
Age (mean)	**32.2 ± 11.4**	**29.12 ± 8.9**	**0.124**
Gender (M/F)	**18/20**	**17/16**	**0.724**
Operation duration (min)	**21.34 ± 8.39**	**24.57 ± 5.87**	**0.049**
Body mass index	21.32 ± 2.38	21.42 ± 2.27	0.713
Length of hospital stay (h)	16.92 ± 5.6	18.03 ± 3.51	0.31
Wound infection	2	4	0.89
Hospital costs ($)	349	205	
Cosmetic results (*n*)			
Excellent	**13**	**16**	**0.287**
Good	**23**	**15**	
Moderate	**2**	**2**	
Bad	**—**	**—**	
